# Acute onset nitrofurantoin‐induced autoimmune hepatitis after urinary tract infection treatment

**DOI:** 10.1002/ccr3.9050

**Published:** 2024-06-11

**Authors:** Ammad Javaid Chaudhary, Muhammad Zarrar Khan, Abdullah Sohail, Syed Murtaza Haider Zaidi, Eric Denha, Deepak Venkat

**Affiliations:** ^1^ Henry Ford Hospital Detroit Michigan USA; ^2^ The University of Iowa Hospitals and Clinics Iowa City Iowa USA; ^3^ King Edward Medical University Lahore Punjab Pakistan; ^4^ Detroit Medical Center/Wayne State University Detroit Michigan USA

**Keywords:** drug‐induced autoimmune hepatitis, elevated liver enzymes, Naranjo adverse reaction, nitrofurantoin toxicity

## Abstract

**Key Clinical Message:**

This case signifies the importance of recognizing DIAIH within the context of antibiotic therapy, especially in older adults and even shortly after common drug exposures for treating UTI.

**Abstract:**

Various drugs can induce immune‐mediated liver damage and in rare instances may lead to autoimmune hepatitis. Here we report an 84‐year‐old woman who developed autoimmune hepatitis less than 3 weeks after treatment for urinary tract infection with the antibiotic nitrofurantoin. She presented with jaundice, right upper quadrant abdominal pain, nausea, and vomiting. In the absence of a history of an autoimmune disorder or elevated liver enzymes in the past; elevated liver enzymes after a short course of Nitrofurantoin and the presence of smooth muscle antibodies strongly suggested autoimmune hepatitis, which was confirmed through biopsy sample analysis. The patient scored 7 points on the Naranjo adverse reaction probability scale. The patient's rapid recovery within 1 month of prednisone therapy supports the association of liver damage with nitrofurantoin use.

## 1 INTRODUCTION

Drug‐induced autoimmune hepatitis (DIAIH) is a rare but serious condition that warrants rapid attention in patients with acute liver injury. Nitrofurantoin, an antibiotic commonly used to treat urinary tract infections (UTI), can induce autoimmune hepatitis (AIH), with the highest occurrence in women (about 85.5% of cases) and in older adults over 65 years (about 38.5% of cases).^1^ Understanding the association between nitrofurantoin and DIAIH is pivotal for early recognition and appropriate management. This case report describes a patient who developed DIAIH within only 3 weeks of a brief course of nitrofurantoin for UTI. This case is particularly noteworthy due to the rapid temporal relationship between nitrofurantoin use and the development of AIH, highlighting the need for heightened awareness of this condition, especially in older women with a history of UTI.

## 2 CASE HISTORY/EXAMINATION

An 84‐year‐old Black/African American woman with a medical history of type 2 diabetes and recurrent UTI presented with postprandial right upper quadrant abdominal pain, nausea, and vomiting. She was currently taking metformin and occasionally took acetaminophen for pain. She also reported taking nitrofurantoin (100 mg) twice daily for 5 days for UTI 18 days before her presentation and denied the use of any herbal remedies or unprescribed supplements. She denied smoking and significant alcohol intake. On examination, she appeared ill, with scleral icterus, jaundice, and guarding of the right upper quadrant to palpation.

## 3 METHODS

**FIGURE 1:**
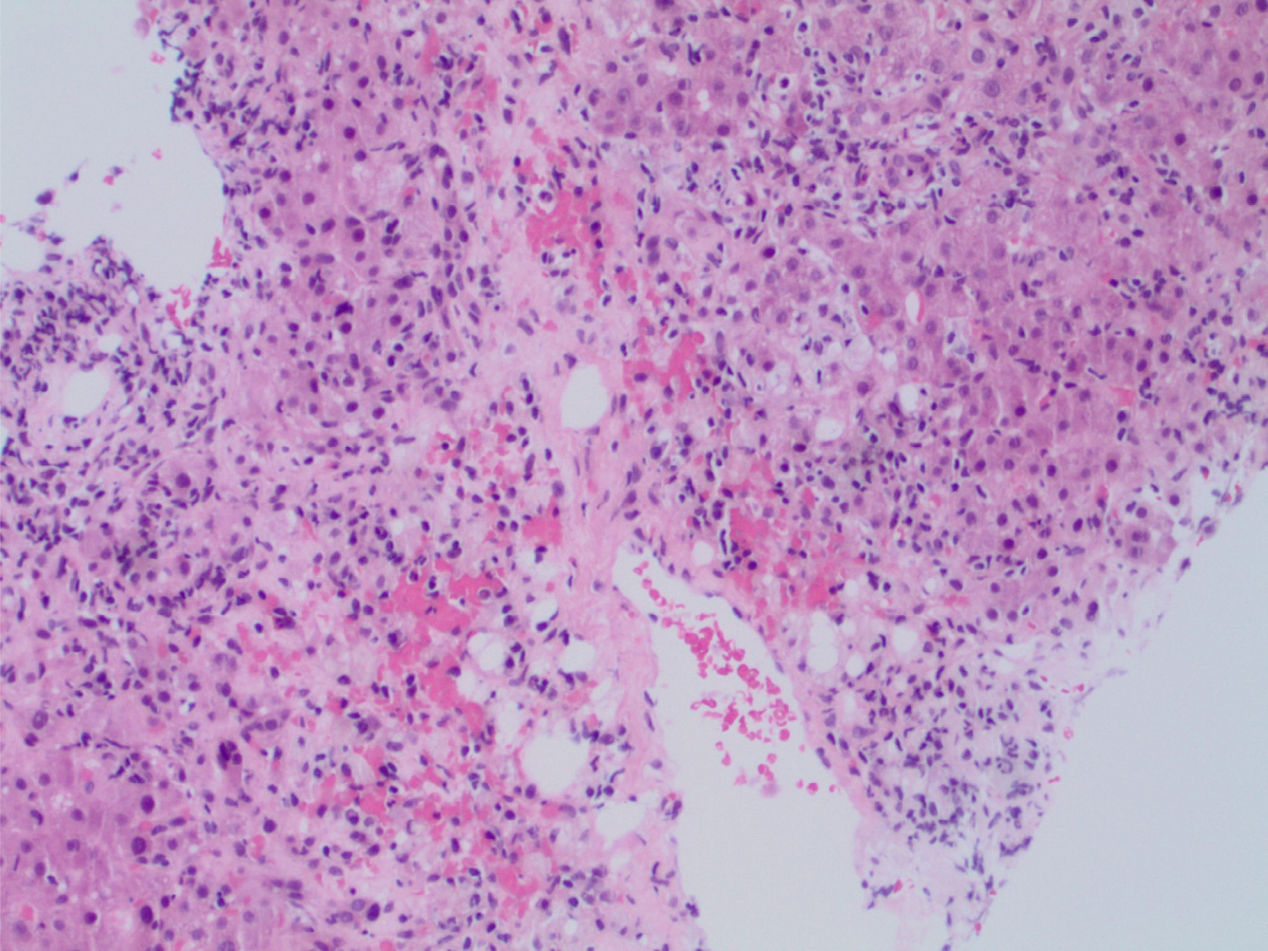
Hepatitis with moderate lymphoplasmacytic necroinflammatory activity.

**FIGURE 2:**
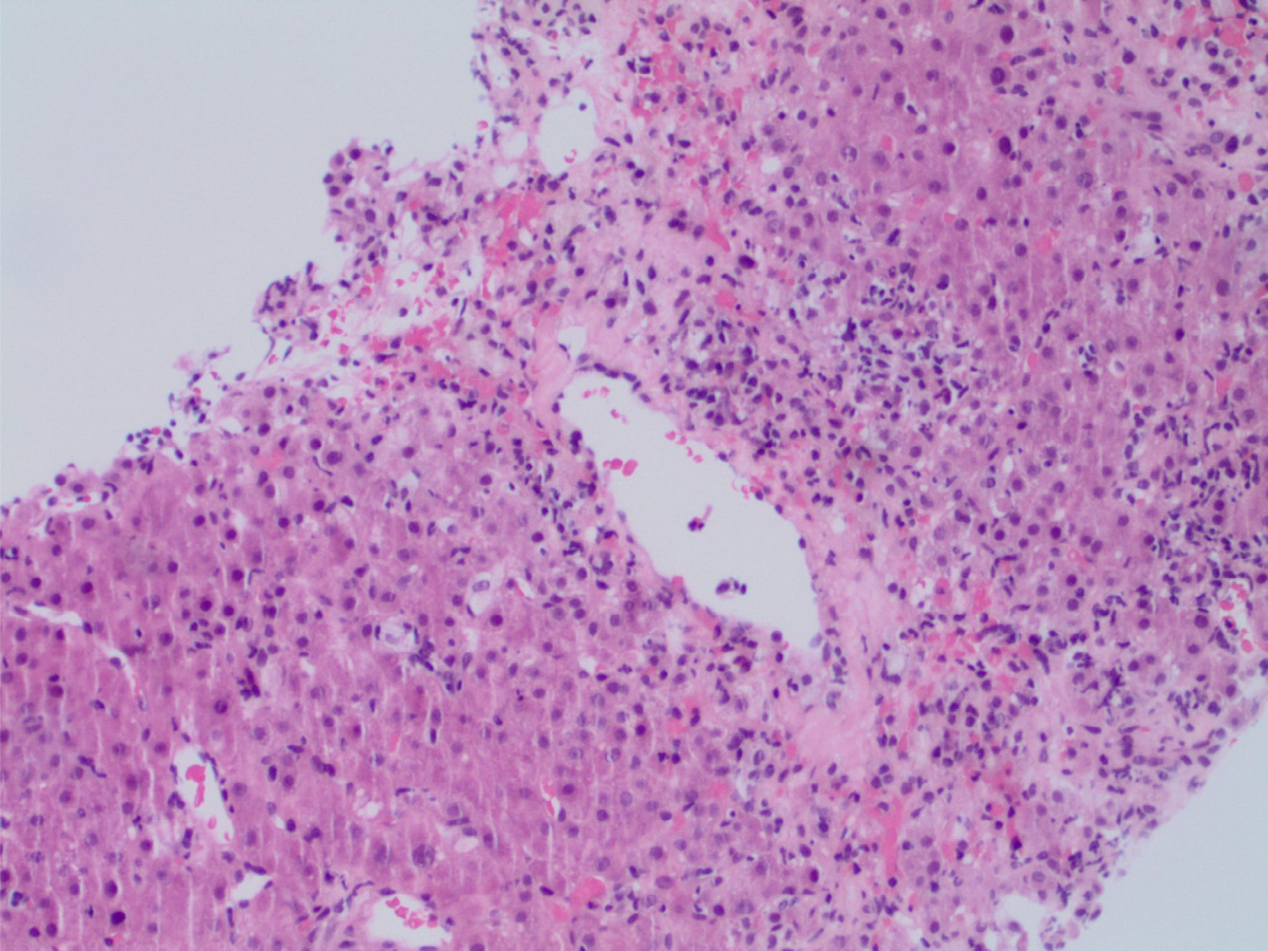
Trichrome special stain reveals focal portal fibrosis present.

**FIGURE 3:**
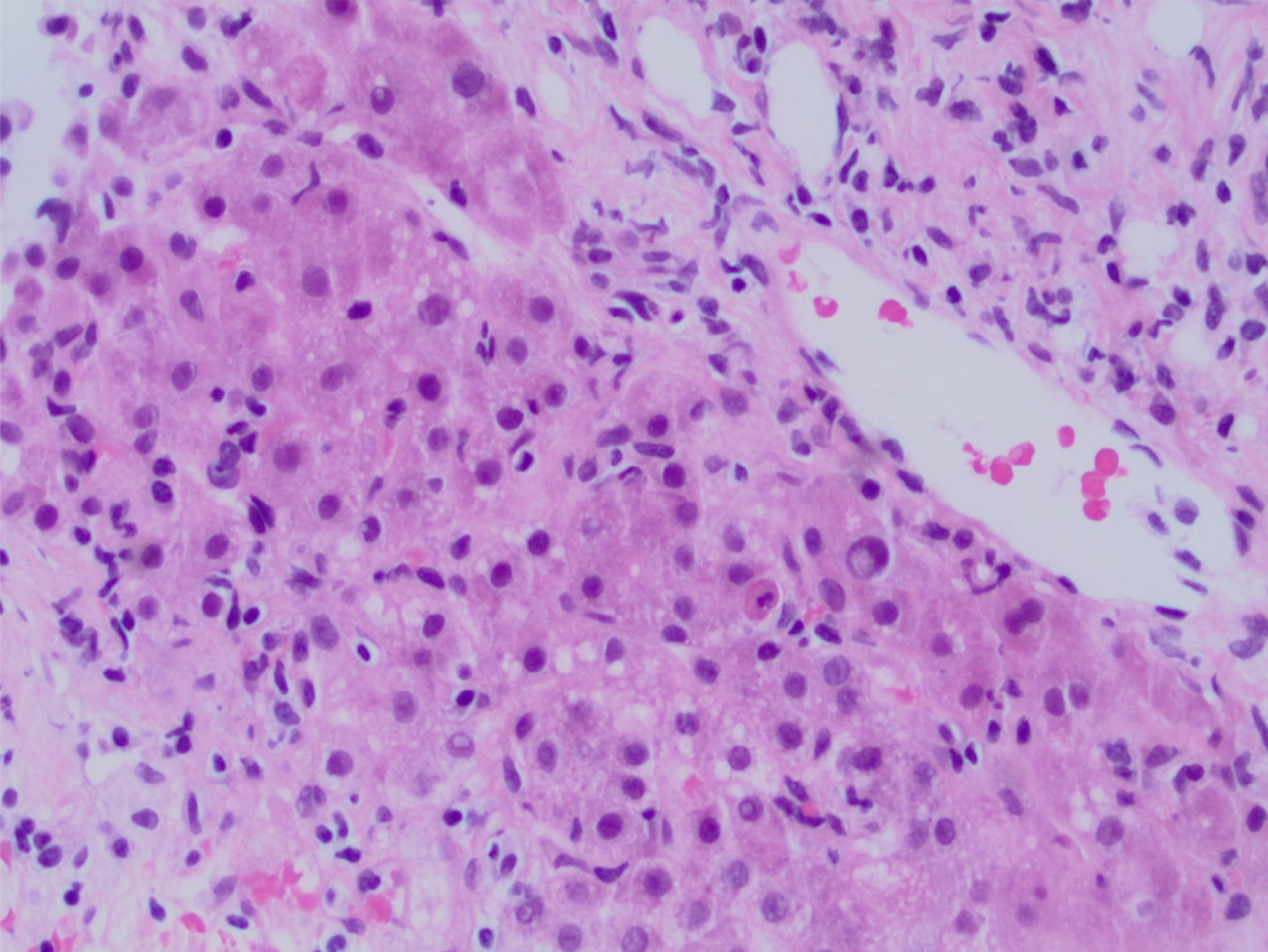
Acute hepatitis with moderate necroinflammatory activity.

Blood tests revealed elevated liver biochemistry with bilirubin 7.6 mg/dL, direct bilirubin 4.4 mg/dL, alanine transaminase 343 IU/L, and aspartate aminotransferase 261 IU/L. Alkaline phosphatase 135 U/L was within the reference range. Coagulation screen, full blood count, electrolytes, and renal function were normal. Serological testing for hepatitis A, B, and C was negative, and toxicology results were negative for acetylsalicylic acid, acetaminophen, and alcohol. Ultrasonography of the liver revealed gallbladder wall thickening with trace pericholecystic fluid and no evidence of gallstones or biliary dilatation. Computed tomography of the abdomen with intravenous contrast showed periportal edema and trace pericholecystic fluid. The autoimmune screen revealed positive smooth muscle antibody (95 U), normal IgA (375 mg/dL), IgG levels (1566 mg/dL), and low IgM (25 mg/dL). Anti‐double‐stranded DNA antibodies, anti‐mitochondrial antibodies, and liver/kidney microsomal antibodies were not detected. Positive smooth muscle antibody results were strongly suggestive of AIH. Upon cessation of nitrofurantoin, liver function tests were still not settling down, and because of the unclear etiology, a liver biopsy was performed, which showed acute hepatitis with moderate necro‐inflammatory activity and trichrome special stain revealed focal portal fibrosis, consistent with DIAIH as shown in Figures 1, 2, 3. The patient scored 7 points on the Naranjo adverse reaction probability scale.

## 4 CONCLUSION AND RESULTS

The patient was started on oral prednisone 40 mg daily. Within 4 weeks, she had an excellent clinical response with a resolution of jaundice and lethargy and improved liver function tests. The prednisone was gradually tapered, and azathioprine was added as a steroid‐sparing agent. This was done based on the guidelines for the use of steroid‐sparing agents after the normalization of LFTs. At 1‐year follow‐up, the patient was solely taking azathioprine and was asymptomatic with a normal liver biochemistry panel.

## 5 DISCUSSION

We present a compelling case of rapid‐onset drug‐induced autoimmune hepatitis occurring 18 days after exposure to a therapeutic dose of nitrofurantoin (100 mg twice daily for 5 days) for an uncomplicated UTI. This case is particularly noteworthy because, upon thorough literature review, no instances have been reported to date of nitrofurantoin‐induced autoimmune hepatitis developing acutely after a brief five‐day course for uncomplicated UTI.

Exposure to certain medications can induce DIAIH, a serious complication that can lead to long‐term liver damage.^2^ Nitrofurantoin, a commonly prescribed antibiotic for uncomplicated UTI, has been implicated as a potential trigger for DIAIH; however, the mechanism underlying this association is not fully understood.^3^ Both short‐term and long‐term nitrofurantoin exposures have been linked to liver damage, with various hepatotoxic patterns. Case studies of DIAIH have included diverse patients who have manifested both acute and chronic liver disease after nitrofurantoin therapy.^4^ Acute injury is most likely to occur within weeks following drug exposure, with hypersensitivity reaction being the most common underlying mechanism.^5^ Additionally, long‐term antibiotic prophylaxis has been linked to chronic autoimmune hepatitis, and in 85% of cases, it appears at least 6 months after initiating treatment.^6^ In many instances, problems do not surface until years into treatment.

In patients exhibiting symptoms of liver damage, differentiating between chronic hepatitis, idiopathic AIH, and DIAIH can be challenging. Notably, histological findings in chronic hepatitis mimic those of idiopathic AIH, with both often including the presence of anti‐nuclear antibodies and anti‐smooth muscle antibodies. DIAIH leads to clinical symptoms that are indistinguishable from idiopathic AIH, such as positive AIH‐related autoantibodies, increased IgG, and similar histology.^7^ Importantly, the presence of advanced fibrosis on histology favors a diagnosis of idiopathic AIH; therefore, when DIAIH is suspected, a liver biopsy should be performed in individuals who are not improving or who have worsening liver injury despite discontinuation of the suspected causative drug.^8^

Extensive literature supports the association between long‐term nitrofurantoin prophylaxis and DIAIH, emphasizing the need for increased vigilance and early recognition of this adverse reaction.^9^, ^10^, ^11^ Nitrofurantoin‐induced DIAIH may result from various mechanisms, including direct hepatotoxicity, immune cell activation, and molecular mimicry between drug metabolites and liver antigens.^12^ It has been hypothesized that nitrofurantoin may act as an antigen, triggering an immune response potentially through an idiosyncratic reaction,^2^ underscoring the importance of considering DIAIH in patients presenting with acute liver injury, even after a short duration of nitrofurantoin therapy. The case described here is unique because of the rapid occurrence of DIAIH following a short course of nitrofurantoin for UTI, which challenges the conventional understanding of nitrofurantoin‐related hepatotoxicity. Our patient's postprandial right upper quadrant abdominal pain, elevated liver enzymes, and positive smooth muscle antibodies supported the diagnosis of AIH, while liver biopsy results showing acute hepatitis with moderate necro‐inflammatory activity and no fibrosis were consistent with drug‐induced liver injury. Importantly, an extensive workup ruled out other likely causes of elevated liver enzymes, and in the absence of a history of autoimmune disorders or prior liver enzyme elevations further strengthened our diagnosis. With normal IgG levels, positive smooth muscle antibodies, and liver biopsy findings supporting the absence of fibrosis, we confidently diagnosed nitrofurantoin‐induced autoimmune hepatitis and initiated an appropriate response with steroids.

Treating patients with DIAIH involves identifying and immediately withdrawing the offending agent, and early recognition is critical for timely, appropriate care. Immunosuppressive therapy with corticosteroids or other agents is often initiated to control immune‐mediated inflammation and prevent progression to chronic liver disease, and this approach worked well for our patient. Although most patients with DIAIH do not require prolonged steroid treatment, some individuals experience elevated liver enzymes after discontinuing steroids. In such cases, prolonged courses of steroids or transition to steroid‐sparing agents, such as azathioprine, may be necessary. Regular monitoring of liver function tests and follow‐up are crucial for assessing the treatment response.^13^

In conclusion, physicians should maintain the possibility of DIAIH on the differential diagnosis for patients with newly developed liver damage and a recent history or current use of nitrofurantoin, and this antibiotic should be discontinued when DIAIH is suspected.

## AUTHOR CONTRIBUTIONS

**Ammad Javaid Chaudhary:** Conceptualization; data curation; formal analysis; investigation; methodology. **Muhammad Zarrar Khan:** Conceptualization; formal analysis; writing – original draft; writing – review and editing. **Abdullah Sohail:** Conceptualization; investigation; methodology; writing – original draft. **Syed Murtaza Haider Zaidi:** Formal analysis; methodology; writing – original draft. **Eric Denha:** Conceptualization; methodology; writing – original draft. **Deepak Venkat:** Supervision; validation; writing – original draft; writing – review and editing.

## FUNDING INFORMATION

This article did not receive any grants.

## CONFLICT OF INTEREST STATEMENT

The authors have no conflict of interest to declare.

## CONSENT

Written informed consent was obtained from the patient to publish this report in accordance with the journal's patient consent policy.

## Data Availability

Data is available upon request from the authors.
